# Editorial: Genomic imprinting and monoallelic gene expression mechanisms and applications

**DOI:** 10.3389/fgene.2022.1067443

**Published:** 2022-11-10

**Authors:** Maria Oczkowicz, Benjamin Tycko, Julie Demars

**Affiliations:** ^1^ National Research Institute of Animal Production, Krakow, Poland; ^2^ Hackensack Meridian Health Center for Discovery and Innovation, Nutley, NJ, United States; ^3^ Institut National de Recherche pour L’agriculture, L’alimentation et L’environnement (INRAE), Paris, France

**Keywords:** imprinting, monoallelic expression, epigenetics, DNA methylation, X chromosome inactivation (XCI)

While this Frontiers series was originally conceived as focusing entirely on genomic imprinting, it evolved to include articles not only on imprinting but also on several related topics in epigenetics, each of which we touch on here. For many years, the principles of inheritance of phenotypic traits, proposed by Mendel (1866), were considered definitive for all genes. However, as the science progressed, it was observed that some genes are regulated differently, with their expression and epigenetic status in offspring dictated by which parent the allele came from (genomic imprinting), or with the expression of one of the alleles randomly extinguished or activated (random monoallelic expression, X-chromosome inactivation). Since the first reports on mammalian genomic imprinting in 1984 (Barton et al., 1984), this phenomenon has been intensively studied—not only in humans and laboratory animal models, but also in economically important domesticated species—to understand mechanisms, biological consequences, and medical implications. Another area has been the study of the evolutionary forces, notably parental conflict (Moore and Haig, 1991), that fostered the emergence of imprinting, which at first glance might appear detrimental to the species’ survival because it can unmask harmful recessive mutations. Many imprinted genes play a crucial role in pre-and post-natal development, shaping physical, intellectual, and psychological development. Likewise, imprinted genes strongly influence farm animals’ performance traits, making such genes good candidates for genetic selection.

While parental conflict is the currently favoured explanation for the emergence of imprinting, in their review article Kaneko-Ishino and Ishino revisit the theory of “defence against the insertion into the host genome” initially proposed by Barlow (1993). They note that germline differentially methylated region (gDMR) insertion events correlate well with the time when a locus gained imprinted regulation, supporting the “host defence” theory. On the other hand, Daigneault, in his review, emphasizes the role of the maternal embryonic factors in regulating epigenetic changes in the paternally transmitted genome early after fertilization and discusses the relationship with male infertility and impaired reproduction in farm animals. In turn, the article by Hubert and Demars discusses the potential use of modern whole genome molecular biology techniques including single-cell RNA-seq, ATAC-seq, Chip-seq etc. in “imprintome” research. The authors also highlight the importance of analysing the interactions of imprinted genes and the complex pathways they create.

Broadening the perspective to medical epigenetics more generally, the latest developments in the use of epigenome profiling methods in clinical human genetics and molecular diagnostics are presented by Mannens et al., who discuss practical applications of DNA methylation (DNAm) arrays and methylation sequencing for diagnosing diseases in which the analysis of the DNA sequence alone can be insufficient. Consistent with a growing appreciation of CpG methylation signatures as a more robust disease classifier than gene expression, it is now possible to diagnose rare diseases and different types of cancers and guide clinical management based on such signatures. Recently, it has been observed that in autism spectrum disorders, abnormalities of methylation on the X chromosome are detected more often than on the other chromosomes. This observation prompted Lasalle to take a closer look at the relationship between the male bias typical of ASD and the inactivation of the X chromosome. In particular, she asked whether anomalies of X inactivation might play a greater role than previously appreciated in the etiology of neurodevelopmental disorders. Along this theme of neurodevelopment, Kong et al., incorporated parent-of-origin effects (POE) into a genome-wide association analysis of behavioural disinhibition (characteristic of many neuropsychiatric disorders) on a large dataset. They identified nine SNPs with significant POE on alcohol dependence or alcohol consumption which could not be detected with standard analysis.

Lastly, Kubasova et al., describe an elegant experiment in which a single hematopoietic stem cell (HSC) proliferates *in vivo*. By analyzing potential random monoallelic gene expression (RME) in this system, the authors could compare their results with prior results from cell lines *in vitro*. Their findings, showing a greatly reduced frequency of RME in the *in vivo* system, are important in the ongoing evaluation of the relative importance of RME *in vivo*.

All this presented work serves to emphasize the importance of genomic imprinting and related epigenetic phenomena both in normal mammalian development and in economic and medical applications. Indeed, the more we know about these processes, the more we are aware of how much remains to be explored.
